# Badomics words and the power and peril of the ome-meme

**DOI:** 10.1186/2047-217X-1-6

**Published:** 2012-07-12

**Authors:** Jonathan A Eisen

**Affiliations:** 1 Genome Center, University of California, Davis, CA, USA; 2 Department of Evolution and Ecology, University of California, Davis, CA, USA; 3 Department of Medical Microbiology and Immunology, University of California, Davis, CA, USA; 4 Center for Population Biology, University of California, Davis, CA, USA; 5 DOE Joint Genome Institute, Walnut Creek, CA, USA

**Keywords:** Genomics, Language, Memes, Omics, Badomics, Genome, Ome-ome, Language parasites

## Abstract

Languages and cultures, like organisms, are constantly evolving. Words, like genes, can come and go–spreading around or going extinct. Here I discuss the spread of one small subset of words that are meant to convey “comprehensiveness” in some way: the “omes” and other words derived from “genome” or “genomics.” I focus on a bad aspect of this spread the use of what I refer to as “badomics” words. I discuss why these should be considered bad and how to distinguish badomics words from good ones.

## Main text

### The rise of genome (the word)

In 1920, “*Verbreitung und Ursache der Parthenogenesis im Pflanzen- und Tierreiche*”–a landmark book by German botanist Hans Winkler–was published [1]. Translating the title into English yields “*Spread and cause of pathogenesis in plant and animal kingdoms*”. An interesting book, no doubt (and one that is available to read online thanks to the Biodiversity Heritage Library [2]), but it is not a fascination with pathogenesis that has kept the book in the limelight for almost 100 years. Instead, it is one passage on page 165 that is critical:

"Ich schlage vor, für den haploiden Chromosomensatz, der im Verein mit dem zugehörigen Protoplasma die materielle Grundlage der systematischen Einheit darstellt den Ausdruck: das Genom zu verwenden und Kerne, Zellen und Organismen, in denen ein gleichartiges Genom mehr als einmal in jedem Kern vorhanden ist, homogenomatisch zu nennen, solche dagegen, die verschiedenartige Genome im Kern führen, heterogenomatisch."

For those not up on their German, the beginning has been translated into English by Joshua Lederberg and Alexa McCray [3]:

"I propose the expression Genom for the haploid chromosome set, which, together with the pertinent protoplasm, specifies the material foundations of the species."

In other words, this was the birth of the term “genome”.

### The spread of the ome-meme

If Winkler were alive today, he would be amazed and what his simple coinage has become. Genomes and “genomics” (the study of genomes)–the concepts and the words–are everywhere and have even spread widely into popular culture. A side effect of this spread has been the proliferation of genomic terminology. In this issue of *GigaScience*, McDonald *et al.*[4] track one aspect of this spread in the emergence of new “ome” words. They describe the collection of omics terms as the “ome-ome”. The main point of their analysis of the ome-ome is that, well, omics is everywhere. And they use this as evidence for the need to develop more standards for, in essence, communication among the different omes (or, well, the tools that deal with the different omes).

The increasing size of the ome-ome suggests (to me at least) that the drive to add “ome” or some variant of it to just about anything is a meme (a spreading cultural practice). Documenting and studying the spread of the ome-meme has become an academic exercise of sorts. And, as with any academic area, they are different camps. Some have approached their analyses in a reserved–perhaps even objective–manner [3]. Yet others have seemed to be almost cheering on the ominess (e.g., [5]). But the majority have been, well, less impressed (e.g., [6-9]).

My above outlining of the studies of the ome-meme focused on articles published in traditional venues (i.e., journals and magazines). But as the world of genomics has changed, so has the world of scientific discourse. And it is on the web where the dissection of the ome-meme is the most extensive. Consider, for example, http://omics.org[10], which both catalogs and has a hierarchical classification of ome terms, or the “-Omes and -omics glossary & taxonomy”, which contains detailed definitions of and references to >100 omes [11].

Of course, the web brings not only traditional web sites like these, but also new fangled things like social media where the ome-meme is a source of much discussion. And much of this social discussion is not so supportive. I should know, as I have become–for better or worse–a hub of much of the critiques, a result of giving out “Awards” such as the “Worst New Omics Word Award” [12] and “Badomics Word of the Day Award” [13]. Examples of some of the “winners” include: sexome, circomics, nascentome, connectome, predatosome, negatome, diseasome, receptorome, uniqueome, drugome, adversomics, bibliome, N-terminome, transactome, nutriome, miRNAome, tRNomics, variome, speechome, vaccinomics, pharmacomicrobiomics, and museomics.

### Why care?

One might ask–rightfully–why do I and others care so much about the spread of omics words? Well, one reason is that attaching “*ome*” in some way to one’s favorite topic does not make that topic genomic-y and does not make that topic more interesting. All it is is marketing. And marketing in science drives me batty.

Another concern is that attaching ome to everything is a form of the overselling of genomics–an issue for which I [14] and others (e.g., [15-17]) have expressed concern. So the spread of the ome-meme, to me, is attaching too much importance to genomics. Mind you, I love genomics. I have been doing it for almost 20 years and never imagine stopping. I think it is a wonderful thing. But it still can be oversold and that can be dangerous.

A third reason that some omics words bother me is that they are clutter. In many cases they are words invented just to allow someone to say they invented a word. They clog up discussions. And with little or no benefit. In a way, one can view many omes as language parasites. They spread by feeding off the strength of other words or concepts. What happens to them, for example, if genomics is no longer “hot”? Will all these ome words seem inane then? Do they stand on their own strength? How can one tell if the ome-meme is parasitizing off the “fitness” of genomics? Well, one hint comes from looking at Figure 1 from McDonald *et al.*[4] The figure shows a pretty clear time point at which the ome-meme took off: 1990. And this just happens to be the year that the human genome project became formalized [18]. While correlation does not equal causation, I for one am comfortable inferring that there is a likely causal relationship here.

**Figure 1:**
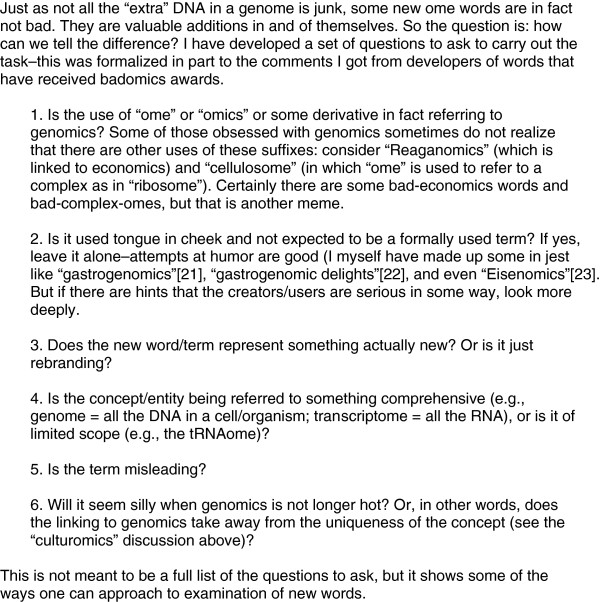
**How to distinguish the badomes from the good ones?**[21-23]

A fourth concern I have with the spread of ome words is that the usage frequently takes away from the uniqueness of some concept by comparing it unnecessarily to genomics. A good example of this is “culturomics”, which was introduced in a brilliant paper on use of high throughput data collection to study of human culture [19]. Sure–what they did had many parallels to genomics and the senior author Erez Lieberman-Aiden wrote me a detailed, elegant, and eloquent justification of why he and others felt that the genomics analogy they were trying to convey was justified. And indeed it was. But to me the analogy should not have extended to the term used to describe their work. This took away from their uniqueness. A similar cheapening of uniqueness has happened repeatedly with other language memes like the ome-meme. Consider for example the proliferation of the suffix “gate” to imply “controversy”. What started with the Nixon Watergate scandal (note: “Watergate” was and still is the name of a building) has led to 100s if not 1000s of “gates”, from “Climategate” to “Monicagate” to “Pepperspraygate” [20]. While William Safire may have get a kick out of adding -gate to everything he wanted to mock in some way, doing so does not make something a controversy any more than adding “ome” makes something genomics. (For help distinguishing badomes from good, see Figure 1)

## Conclusions

Genomics is a wonderful topic. And it has great potential value. But adding “ome” or “omics” onto some term does not suddenly make it “genomic-y”. The power of genomics does not simply transfer with a suffix. In addition, new concepts do not need to latch onto the ome-meme if they are strong and interesting in and of themselves. Comparisons to genomics can be very useful, but including genomics in some way in the term itself is potentially unwise.

In my youth (graduate school), I coined an omics word that was not done tongue in cheek: phylogenomics. My original usage was a bit narrow and probably could get a #badomics award. But fortunately I and others reworked the term to be broader and cleaner–in essence it is now used to refer to the integration of phylogenetics and genomics. Words are not owned by anyone. Badomics words can become decent–even good. If you think there is a need for a new omics word–by all means–put it out there. But don’t be a casual vector for the spread of the ome-meme: give at least a few thoughts to whether the word is useful and necessary. And maybe you might even get a “good omics word award.”

## Competing interests

The author declared that they have no competing interest.

## Authors’ contributions

JAE did everything.

## Authors’ information

JAE is a Professor at the University of California, Davis, with appointments in the Genome Center, the Department of Medical Microbiology and Immunology, the Department of Evolution and Ecology, and the Center for Population Biology. In addition, he holds an Adjunct Appointment at the DOE Joint Genome Institute. Prior to moving to Davis, he was on the faculty at The Institute for Genomic Research (TIGR) for eight years. He is also the Academic Editor in Chief of PLoS Biology, a science blogger (e.g. http://phylogenomics.blogspot.com) and microblogger (http://twitter.com/phylogenomics), and is a bit obsessed both with the use of the ome-meme and with open access publishing.
